# Advances in the genetics of refractive errors: Contributions from the CREAM consortium

**DOI:** 10.1111/aos.70025

**Published:** 2025-11-13

**Authors:** Sze Wai Rosa Li, Xi He, Louise Terry, Virginie J. M. Verhoeven, Samantha Sze‐Yee Lee, Gareth Lingham, Jeremy A. Guggenheim, David A. Mackey, Seang‐Mei Saw, Caroline C. W. Klaver, Chi Pui Pang, CREAM Consortium

**Affiliations:** ^1^ School of Medicine Cardiff University Cardiff UK; ^2^ School of Optometry & Vision Sciences Cardiff University Cardiff UK; ^3^ Department of Clinical Genetics, Erasmus MC University Medical Center Rotterdam Rotterdam The Netherlands; ^4^ Department of Ophthalmology, Erasmus MC University Medical Center Rotterdam Rotterdam The Netherlands; ^5^ Lions Eye Institute, Centre for Ophthalmology and Visual Science University of Western Australia Perth Western Australia Australia; ^6^ Singapore Eye Research Institute Singapore National Eye Centre Singapore Singapore; ^7^ Ophthalmology & Visual Sciences Academic Clinical Program (Eye ACP) Duke‐NUS Medical School Singapore Singapore; ^8^ Saw Swee Hock School of Public Health National University of Singapore Singapore Singapore; ^9^ Department of Epidemiology, Erasmus MC University Medical Center Rotterdam Rotterdam The Netherlands; ^10^ Department of Ophthalmology Radboud University Medical Center Nijmegen The Netherlands; ^11^ Institute of Molecular and Clinical Ophthalmology University of Basel Basel Switzerland; ^12^ Department of Ophthalmology and Visual Sciences The Chinese University of Hong Kong Hong Kong Hong Kong; ^13^ Hong Kong Hub of Paediatric Excellence The Chinese University of Hong Kong Hong Kong Hong Kong; ^14^ Joint Shantou International Eye Center of Shantou University and The Chinese University of Hong Kong Hong Kong Hong Kong

**Keywords:** genetics, genome‐wide association study, myopia, refractive error, whole exome sequencing

## Abstract

The Consortium for Refractive Error and Myopia (CREAM) was established in 2011, bringing together an international team of researchers studying more than 30 cohorts. Since its establishment, CREAM has played a pivotal role in research investigating the genetics of myopia and other refractive errors, serving as a key driver of progress in the field. The aim of this review is to highlight the latest advances and insights from CREAM, with a focus on research carried out in the past 5 years. We performed a literature review of journal articles authored by the CREAM consortium since the year 2020, when the last review of CREAM consortium findings was published. Key discoveries from recent CREAM studies were the identification of *SIX6*, *CRX*, *PER3*, *PDCD6IP*, *MAPT*, *CHST6*, *GRHL2*, *USH2A*, *P4HTM*, *COL4A4* and *ATM* as high‐confidence candidate genes associated with myopia development. Variants in enhancers and lncRNA regions were shown to have potential regulatory effects on refractive error; the *DDIT4* gene was highlighted as a potential hotspot for future analyses. A polygenic risk score for predicting high myopia with an area under the curve (AUC) accuracy of 0.78 was made openly available; prediction accuracy was close to that required for clinical use. A shared genetic architecture for refractive error and axial length was confirmed. Novel findings were the identification of rare, large‐effect gene variants through targeted and whole exome sequencing and the development of a polygenic risk score for predicting children at risk of developing high myopia. Large‐scale multi‐ancestry genome‐wide association studies of the myopia endophenotypes axial length and corneal curvature doubled the number of common genetic variants known to be associated with these traits. Nevertheless, much remains to be done to fulfil the promise of myopia genetics research for improving the detection of children at above‐average risk of high myopia, and the prevention and treatment of myopia.

## 1 INTRODUCTION

Myopia, commonly known as ‘near‐sightedness’, is a vision disorder that is estimated to affect 2.6 billion people worldwide. Current forecasting suggests the prevalence of myopia will increase to 5.0 billion people (50% of the global population) by 2050 (Holden et al., 2016). Typically, myopia is caused by excessive axial elongation of the eye. This axial elongation of myopic eyes is an important risk factor for eye diseases such as glaucoma, retinal detachment and myopic macular degeneration (Haarman et al., 2020; Ohno‐Matsui et al., 2021).

Both genetics and lifestyle factors such as reduced time spent outdoors are implicated in the pathogenesis of myopia (Morgan et al., 2021; Tedja et al., 2019). Advances in molecular genetics over the past two decades have considerably expanded our knowledge of the genetic contribution to refractive errors. A major finding has been that myopia and hyperopia are associated with a complex array of different gene variants, most of which have very subtle effects. Despite intensive research efforts, molecular genetic findings have been able to explain less than one‐fifth of the heritability of refractive error (Hysi et al., 2020; Tedja et al., 2018, 2019). This underscores the challenging nature of uncovering gene variants of small effect and discovering rare, large‐effect mutations within this field. Further research into the genetics of myopia is needed to understand why the heritability of refractive error is so high despite the unquestionably important role of environmental risk factors for myopia.

## 2 THE CREAM CONSORTIUM

The Consortium for Refractive Error and Myopia (CREAM) is an international research collaboration that was established in 2011 with the aim of increasing knowledge about the genetics of refractive error and myopia. The consortium brought together researchers studying 37 different cohorts from across the world (Table 1). Each study cohort provided valuable genetic and phenotypic information, predominantly in populations of European and East Asian ancestry. CREAM's collaborative approach facilitated large genome‐wide association study (GWAS) meta‐analyses to be carried out. These GWAS meta‐analyses have played a crucial role in driving the identification of novel myopia genes (Tedja et al., 2019). In 2020, the CREAM consortium summarized its initial contribution to myopia research in a review article (Haarman, Tedja, et al., 2021). In the current article, we review the CREAM consortium's findings during the period from 2020 to 2025. However, we begin with a brief summary of the pre‐2020 findings. A list of abbreviations is given in Table 2.

**TABLE 1:** Study cohorts contributing to the CREAM Consortium. Reproduced from supplementary table 1a of Tedja et al. (2018).

Study number	Study name	Origin	Study design	*N*
*European cohorts*				
1	1958 British Birth Cohort	The United Kingdom	Population‐based study	1658
2	ALIENOR	France	Population‐based study	509
3	ALSPAC‐Mothers	The United Kingdom	Population‐based study	1865
4	ANZRAG	Australia	POAG cases	648
5	AREDS	The United States	Population‐based study	1842
6	BATS	Australia	Population‐based twin study	158
7	BMES	Australia	Population‐based study	1896
8	Croatia‐Korcula	Croatia	Population‐based study	822
9	Croatia‐Split	Croatia	Population‐based study	344
10	Croatia‐Vis	Croatia	Population‐based study	527
11	DCCT	The United States	Clinical trial	791
12	EGCUT	Estonia	Population‐based study	904
13	EPIC‐Norfolk	The United Kingdom	Population‐based study	1084
14	ERF	The Netherlands	Family‐based study	2610
15	FECD	The United States	Case–control*	393
16	FITSA	Finland	Population‐based twin study	329
17	Framingham	The United States	Population‐based study	2729
18	Gutenberg Health Study 1	Germany	Population‐based study	2738
19	Gutenberg Health Study 2	Germany	Population‐based study	1140
20	KORA	Germany	Population‐based study	2372
21	OGP Talana	Italy	Population‐based study	509
22	ORCADES	The United Kingdom	Population‐based study	1165
23	Rotterdam Study I	The Netherlands	Population‐based study	5787
24	Rotterdam Study II	The Netherlands	Population‐based study	2038
25	Rotterdam Study III	The Netherlands	Population‐based study	2950
26	TEST	Australia	Population‐based twin study	267
27	Twins UK	The United Kingdom	Population‐based twin study	4342
28	WESDR	The United States	Case–control from population‐based study	295
29	YFS	Finland	Population‐based study	1480
Total *N*				44 192
*Asian cohorts*				
30	Beijing Eye Study	China	Population‐based study	590
31	Nagahama	Japan	Population‐based study	2730
32	SCES	Singapore (Chinese)	Population‐based study	1724
33	SIMES	Singapore (Malay)	Population‐based study	2275
34	SINDI	Singapore (Indian)	Population‐based twin study	2110
35	SP2‐1M	Singapore	Population‐based study	818
36	SP2‐610	Singapore	Population‐based study	871
37	STARS PARENT	Singapore	Population‐based study	817
Total *N*				11 935

**TABLE 2:** A list of acronyms used in this literature review.

AL	Axial length
AOSW	Age‐of‐onset of spectacle wear
BDES	Beaver Dam Eye Study
CC	Corneal curvature
GWAS	Genome‐wide association studies
MMD	Myopic macular degeneration
ncRNA	Non‐coding RNA
pLOF	Putative loss of function
SER	Spherical equivalent
SNP	Single nucleotide polymorphism
WES	Whole exome sequencing

## 3 SUMMARY OF CREAM RESEARCH STUDIES 2011–2020

CREAM's first collaborative work replicated a previously identified genetic locus on chromosome 15, where the *GJD2* gene is located (Haarman, Enthoven, et al., 2021; Haarman, Tedja, et al., 2021; Verhoeven et al., 2012). *GJD2* codes for the protein connexin‐36, which forms gap junctions between cones, rods and bipolar cells that serve as electrical synapses for retinal signal transduction (Brown‐Panton et al., 2023; Haarman, Enthoven, et al., 2021; Haarman, Tedja, et al., 2021; Quint et al., 2021; van der Sande et al., 2022). The first CREAM GWAS for refractive error identified 24 novel loci (Verhoeven et al., 2013) and replicated many of the genetic variants identified in a GWAS for age of onset of spectacle wear for myopia carried out by the direct‐to‐consumer genomics company, 23andMe (Kiefer et al., 2013). By increasing the number of collaborating institutions, a subsequent CREAM GWAS meta‐analysis that included 160 420 participants (with independent replication in an additional 95 505 participants) increased the number of validated refractive error loci to 161 (Tedja et al., 2018). A polygenic score derived from these genome‐wide significantly associated variants demonstrated a 40‐fold higher risk of myopia (more negative refractive error) for individuals in the first decile of the polygenic score compared to those in the tenth decile (Tedja et al., 2018). This work further suggested that cellular processes of cell–cell adhesion, synaptic transmission, calcium ion binding and cation channel activity were linked to the development of myopia and identified many genes related to the cell cycle and growth pathways, such as the MAPK and TGF‐beta/SMAD pathways. Other key findings included the identification of genes involved in retinal cell physiology and light processing (*RGR*, *RP1L1*, *RORB* and *GNB3*); confirmation of the role of the TGF‐beta signalling pathway in myopia; evidence of dopamine pathway involvement through the *DRD1* gene; and novel mechanisms, such as rod‐and‐cone bipolar synaptic neurotransmission, anterior‐segment morphology and angiogenesis (Tedja et al., 2018).

The largest GWAS meta‐analysis performed by the CREAM consortium was reported by Hysi et al. (2020). In this study of 542 934 participants of European ancestry, Hysi et al. identified 449 genetic loci independently associated with refractive error, of which 336 were novel. Post‐GWAS analysis suggested that molecular processes such as regulation of intraocular pressure, structural development of the eye and circadian rhythms contributed to myopia. Retinal genes were highly represented, including *TRPM1* and *VSX2*, which play key roles in rod ON bipolar cells and cone bipolar cells, respectively. A polygenic score composed of the 890 most strongly associated single nucleotide polymorphisms (SNPs) explained 12% of the variation in refractive error in an independent sample of adults from the EPIC‐Norfolk cohort; quantifying prediction accuracy using the area under the receiver operating characteristic curve (AUROC), the polygenic score predicted high myopia (≤−5.00 D) with an accuracy of AUROC = 0.75 in the EPIC‐Norfolk cohort (Hysi et al., 2020).

The early CREAM studies supported a wide range of research initiatives by independent groups. Highlights included the discovery of the association of the *NRXN1* gene with refractive astigmatism (Li et al., 2015), gene–environment interactions at novel loci such as *AREG*, *GABRR1* and *PDE10A* in Asian populations (Fan, Verhoeven, et al., 2016) and preliminary evidence of interaction with near work for 39 SNPs derived from a CREAM meta‐GWAS, with the strongest signal at *ZMAT4* (Fan, Guo, et al., 2016). Similarly, eight novel genome‐wide significant loci (*RSPO1*, *C3orf26*, *LAMA2*, *GJD2*, *ZNRF3*, *CD55*, *MIP* and *ALPPL2*) associated with axial length were identified (Cheng et al., 2013). GWAS analyses conducted by the CREAM consortium also resulted in the replication of previously reported gene variants associated with corneal astigmatism, such as *PDGFRA*, *ZC3H11B*, *GJD2*, *RASGRF1* and *RFBOX1*, while validating other promising candidate genes and strengthening the evidence for their role in refractive error development (Li et al., 2015; Shah et al., 2018). Other notable achievements included the evidence of no causal association between higher serum vitamin D levels and myopia based on Mendelian randomization analysis, which suggested that the protective effect of time spent outdoors against myopia was not mediated via vitamin D (Cuellar‐Partida et al., 2017).

## 4 REVIEW SUMMARY OF CREAM RESEARCH STUDIES 2020–2025

In the past 5 years, CREAM studies have shifted attention to rare variants and non‐coding regions of the genome, which had been neglected before due to technological limitations (Table 3).

**TABLE 3:** Key points: an overview of main findings identified across papers reported during 2020–2025 that were included within this review, highlighting key insights into myopia development.

1	In general, the same genetic variants predispose individuals to high myopia, low myopia and hyperopia.
2	Polygenic scores have been developed to predict which children are at greatest risk of developing high myopia in the future; the performance of these polygenic scores is approaching the level required for use in the clinic. However, performance varies greatly depending on the ancestry of the patient.
3	A very wide variety of genetic mechanisms are implicated in conferring susceptibility to myopia, including non‐coding enhancers, microRNAs and lncRNAs.
4	Exome sequencing studies have begun to identify novel rare variants associated with myopia, sometimes within genes previously identified by GWAS to be associated with refractive error.
5	An exome‐array study identified 129 genes associated with refractive error, of which only 10 were previously identified to be associated with refractive error.
6	A recent GWAS for corneal curvature identified 20 novel loci.
7	A multiethnic GWAS study of axial length identified 5 were novel, not previously reported in studies biased towards participants of European ancestry.

## 5 RARE VARIANTS IDENTIFY HIGH‐CONFIDENCE MYOPIA‐ASSOCIATED GENES

Genetic variants identified through GWAS meta‐analyses account for about 12%–19% of the inter‐subject variation in refractive error (Clark et al., 2023; Hysi et al., 2020). However, twin and family heritability studies suggest that genetics accounts for 50%–80% of the inter‐subject variation in refractive error (Sanfilippo et al., 2010). This gap, known as the ‘missing heritability’, could be attributed to several factors, one of which is the presence of rare variants (typically defined as variants with a minor allele frequency < 1%). The advance of whole exome sequencing (WES), whole genome sequencing (WGS) and exome array genotyping techniques now permit accurate, cost‐effective genotyping of rare variants. Earlier GWAS analyses ignored these rare variants, due to limitations of accurate genotyping using SNP arrays and imputation. Rare variants often have serious adverse impacts on gene function, resulting in a large phenotypic effect in patients carrying such a variant. Thus, rare variants offer a direct route to identifying high‐confidence candidate myopia genes; by contrast, it is typically uncertain which nearby gene or genes are impacted by the SNPs identified in GWAS analyses.

Using the gene‐based SKAT‐O algorithm, which aggregates the effects of rare variants in each gene, Patasova et al. (2022) reported that the *SIX6* and *CRX* genes were significantly associated with refractive error in individuals of European ancestry. The association of rare variants in *CRX* with refractive errors in this study was noteworthy because *CRX* is known to be important in photoreceptor development (Furukawa et al., 1997). Furthermore, mutations in *SIX6* and *CRX* are associated with retinal dystrophies such as Leber congenital amaurosis, cone‐rod dystrophy and also with microphthalmia (Aldahmesh et al., 2013; Freund et al., 1998; Swain et al., 1997). Indeed, it is now recognized that a wide range of retinal dystrophy disease genes can cause early onset high myopia (Wang et al., 2025). Patasova et al. concluded that rs146737847 and rs61748438 were the lead SNPs driving the association in the *SIX6* and *CRX* gene regions, respectively. The discovery that rare variants can play significant roles in refractive errors and myopia has since been supported by several studies from the Myopia Associated Genetics Intervention Consortium (MAGIC) (Chen et al., 2024; Yuan et al., 2024). Patasova et al. also considered *PRSAP52*, *PCCA*, *MIR4683*, *SELENOM*, *NAPA* and *VWA8* as suggestive significant findings. Further replication in additional cohorts supported the association of *NAPA* with refractive error (Patasova et al., 2022).

In a separate study focussing on rare variants genotyped using exome arrays, Musolf et al. (2023) analysed a multi‐ancestry discovery cohort of 13 000 CREAM participants along with a replication sample of 14 000 participants, finding a total of 129 candidate gene associations driven by rare variants. More than half of these genes (66/129) were already known to be associated with an ocular phenotype; however, the link to refractive error was a novel discovery for most of the genes. To improve power and robustness, the study by Musolf et al. employed three different methods (EMMAX‐VT, EMMAX‐CMC and ACAT) to test for an association with refractive error. Only rare variants were included in the analyses. Four genes (*CDF15*, *PDCD6IP*, *RRM2* and *ST6GALNAC5*) were co‐identified in meta‐analyses of multi‐ancestry cohorts across different methods, three of which overlapped with the findings in Indo‐European ancestry cohorts (*PDCD6IP* being replaced by *PIK3CA* in this ancestry group). *GSTM5* and *WEE1* were uniquely identified in East Asian cohorts. Among all of the identified genes, seven (*PER3*, *PDCD6IP*, *MAPT*, *CHST6*, *GRHL2*, *USH2A* and *P4HTM*) were prioritized as high‐confidence candidate genes based on their internal replication evidence, expression in human retina, relevance to ocular phenotypes and previous GWAS evidence. Eighteen potential missense causal variants were highlighted by wANNOVAR analysis. Variant rs201527662 in *USH2A* was annotated as a ‘damaging’ variant by all prediction algorithms and had the highest CADD score = 36. This rare variant was associated with high myopia and was distinct from the known *USH2A* variants associated with retinitis pigmentosa. Musolf et al. further performed a sensitivity analysis in which a polygenic score was included as a covariate when testing for rare variant associations, in order to control for potential bias from common variants. The results were robust to the inclusion of the polygenic score, confirming that common variants were not driving the association.

CREAM also carried out a large‐scale single‐variant GWAS for refractive error using WES data from UK Biobank participants, as reported by Guggenheim et al. (2022). The GWAS focused on 28 340 rare putative loss‐of‐function (pLOF) variants and 468 011 rare missense variants, which were expected to have major functional impacts on the phenotype. Unexpectedly, despite a sample size of 51 624 participants, none of the rare pLOF variants were associated with refractive error. However, two rare missense variants were significantly associated with refractive error: rs146737847, which results in a p.Glu129Lys substitution in the *SIX6* gene, and rs61748438, which causes a p.Val66Ile substitution in the *CRX* gene. Although *SIX6* and *CRX* had been previously implicated in myopia development, the rare missense variants were found to exhibit much larger effects. Fine mapping suggested that an additional novel missense variant rs33912345 in the *SIX6* gene was also independently associated with refractive error. Sixteen common missense variants, located in 12 distinct regions, were also revealed to have a significant association with refractive error. Putative causal variants were identified in *PDE11A*, *COL4A4*, *PRSS56*, *BMP3*, *RASGEF1B*, *RGR*, *KAZALD1*, *ARMS2*, *ATM*, *GNB3*, *GSDMA*, *GNGT2* and *ZNF652*. Of these genes, seven were novel findings (Table 4). In independent validation analyses, 9 of the 19 putative causal variants found in the discovery phase were successfully replicated after correction for multiple testing, and a further five variants reached nominal significance. In contrast to the CREAM rare variant analysis of Musolf et al. (2023), the rare variant‐associated genes prioritized in the study of Guggenheim et al. had a high overlap with genes identified through common variant GWAS. Further work will be required to reveal whether the overlap of regions harbouring rare variants and common variants associated with refractive error is the exception or the norm.

**TABLE 4:** Summary of novel gene variants identified in CREAM studies since 2020.

Gene	rsID	Associated trait	Ancestry group	Replication cohort	Study
*DDIT4*	—	SER + myopia	European + Asian	Generation R	Tedja et al. (2025)
*ANAPC16 (hs1834)*	—	SER + myopia	European + Asian	Generation R	Tedja et al. (2025)
*SIX6*	rs33912345	SER + AOSW	European	UK Biobank	Guggenheim et al. (2022)
	rs146737847	SER	European	CREAM + BDES	Patasova et al. (2022)
*CRX*	rs61748438	SER + AOSW	European	UK Biobank	Guggenheim et al. (2022)
	rs61748438	SER	European	CREAM + BDES	Patasova et al. (2022)
*COL4A4*	rs2229814	SER + AOSW	European	UK Biobank	Guggenheim et al. (2022)
*RASGEF1B*	rs1077803	SER + AOSW	European	UK Biobank	Guggenheim et al. (2022)
*ARMS2*	rs10490924	SER	European	—	Guggenheim et al. (2022)
*ATM*	rs672655	SER	European	—	Guggenheim et al. (2022)
*GSDMA*	rs7212938	SER	European	—	Guggenheim et al. (2022)
*GNGT2*	rs35638197	SER + AOSW	European	UK Biobank	Guggenheim et al. (2022)
*ZNF652*	rs2072153	SER + AOSW	European	UK Biobank	Guggenheim et al. (2022)
*BMP4*	rs534912805	SER + AOSW	European	UK Biobank	Guggenheim et al. (2022)
	rs17563				
*USP1*	rs945170	CC	European + Asian	UK Biobank	Fan et al. (2020)
*FNDC3B/GHSR*	rs485554	CC	European + Asian	UK Biobank	Fan et al. (2020)
*SOX2*	rs10663094	CC	European + Asian	UK Biobank	Fan et al. (2020)
*LCORL*	rs16896276	CC	European + Asian	UK Biobank	Fan et al. (2020)
*RP11‐125O18.1*	rs6831679	CC	European + Asian	UK Biobank	Fan et al. (2020)
*OFCC1*	rs67612840	CC	European + Asian	UK Biobank	Fan et al. (2020)
*U6*	rs961755	CC	European + Asian	UK Biobank	Fan et al. (2020)
*NHSL1*	rs4620141	CC	European + Asian	UK Biobank	Fan et al. (2020)
*RP11‐91P17.1*	rs7004112	CC	European + Asian	UK Biobank	Fan et al. (2020)
*LMX1B*	rs483714	CC	European + Asian	UK Biobank	Fan et al. (2020)
*FRAT1/FRAT2/ ARHGAP19*	rs10786330	CC	European + Asian	UK Biobank	Fan et al. (2020)
*IGF2*	rs7948458	CC	European + Asian	UK Biobank	Fan et al. (2020)
*ADAMTS20*	rs11181913	CC	European + Asian	UK Biobank	Fan et al. (2020)
*SNORD36*	rs9316971	CC	European + Asian	UK Biobank	Fan et al. (2020)
*INTS6*	rs7327381	CC	European + Asian	UK Biobank	Fan et al. (2020)
*STON2*	rs4083463	CC	European + Asian	UK Biobank	Fan et al. (2020)
*FBN1*	rs9806595	CC	European + Asian	UK Biobank	Fan et al. (2020)
*ADAMTS7*	rs4887113	CC	European + Asian	UK Biobank	Fan et al. (2020)
*MAPRE2*	rs184926585	CC	European + Asian	UK Biobank	Fan et al. (2020)
*BMP7*	rs6064518	CC	European + Asian	UK Biobank	Fan et al. (2020)
*SLC25A12*	rs57718990	AL	Non‐Hispanic white, Hispanic/Latino, East‐Asian, African American	CREAM subset	Jiang et al. (2023)
*BMP3*	rs1353386	AL	Non‐Hispanic white, Hispanic/Latino, East‐Asian, African American	CREAM subset	Jiang et al. (2023)
*RGR*	rs140405296	AL	Non‐Hispanic white, Hispanic/Latino, East‐Asian, African American	CREAM subset	Jiang et al. (2023)
*RBFOX1*	rs58514548	AL	Non‐Hispanic white, Hispanic/Latino, East Asian, African American	CREAM subset	Jiang et al. (2023)
*MYO5B*	rs55754534	AL	Non‐Hispanic white, Hispanic/Latino, East Asian, African American	CREAM subset	Jiang et al. (2023)
*PRSS56*	—	AL	Non‐Hispanic white	—	Jiang et al. (2023)

Abbreviations: AL, axial length; AOSW, age‐of‐onset of spectacle wear; BDES, Beaver Dam Eye Study; CC, corneal curvature; SER, spherical equivalent.

## 6 NOVEL VARIANTS IN ncRNAs AND ENHANCERS LINKED TO MYOPIA

Alongside protein‐coding genes, non‐coding RNAs (ncRNAs) and non‐coding genomic regions are emerging as key elements in the genetic landscape of myopia (Swierkowska et al., 2022). Recent GWAS have identified numerous risk loci within non‐coding regions, which suggest a regulatory role in ocular development (Li et al., 2022; Tedja et al., 2018; Wang et al., 2022). ncRNAs, including microRNAs and long ncRNAs, are thought to modulate gene expression involved in eye growth and refractive error pathways. Although an increasing number of genes associated with eye growth are being identified, the specific contributions of non‐coding regions to myopia pathogenesis remain incompletely understood.

Leveraging summary statistics of a CREAM consortium GWAS analysis of 160 420 participants, the first large‐scale study of ncRNAs and enhancers for refractive error and myopia was reported by Tedja et al. (2025). The results indicated that two SNPs located within distinct microRNAs were associated with myopia, as were 54 SNPs located in the binding sites of 36 different microRNAs, 19 SNPs situated in 14 different enhancer regions and 103 SNPs located in 81 IncRNA regions. The target and host genes of these ncRNAs and enhancers showed an over‐representation in human retinal cells. Interestingly, this study also reported overlaps of an enhancer region, a microRNA binding site and an lncRNA in the vicinity of *DDIT4*, linking this gene with myopia for the first time. *DDIT4* is an mTOR pathway inhibitor that plays a role in neuronal differentiation, migration and neuronal cell death. To evaluate the cumulative effects of these regulatory variants, Tedja et al. derived a genetic risk score of SNPs situated in miRNAs, miRNA binding sites, lncRNAs and enhancer regions. The genetic risk score was significantly associated with refractive error and axial length progression in a sample of Dutch children aged 9–13 years. The highest‐ranked candidate variants in enhancer regions were linked to plausible retina‐to‐sclera cascades, including *BMP4* with eye development, *DDIT4‐NTM* with neurogenesis, *DNAJB12* with photoreceptor metabolism, *VIPR2* with neural signalling and *ANAPC16* with TGF‐beta signalling. In summary, the study of Tedja et al. (2025) suggested that enhancer regions and lncRNAs are especially important in myopia development. The authors recommended future analyses to investigate elements such as silencers and insulator regions.

## 7 SHARED GENETIC CONTRIBUTION TO DIFFERENT TYPES OF REFRACTIVE ERROR AND TO OCULAR COMPONENT DIMENSIONS

Refractive error spans the spectrum from high myopia, through emmetropia, to high hyperopia. In view of their different clinical presentations, a fundamental question in myopia research is the extent to which genetic susceptibility is shared between low myopia and high myopia, and indeed between myopia and hyperopia. Tideman et al. (2021) conducted separate GWAS analyses for four different case–control samples: high myopia versus emmetropia; low myopia versus emmetropia; hyperopia versus emmetropia and low myopia versus hyperopia. They found evidence that genetic risk was shared across all four GWAS analyses and across European and Asian participants. While acknowledging that high myopia can be caused by inheriting a rare, pathogenic variant or through environmental risk factors, the authors suggested that genetic risk variants predisposing to low myopia were over‐represented in patients with high myopia, implying that many patients with high myopia carry a large burden of low‐effect variants.

Apart from refractive error, myopia‐related traits, including corneal curvature and axial length, have also been investigated in CREAM studies. A shared genetic background of refractive error and the corneal curvature and axial length endophenotypes has been confirmed. Corneal curvature determines two‐thirds of the refractive power of the eye. Fan et al. (2020) conducted the largest trans‐ethnic GWAS of corneal curvature published to date, examining a sample of 29 580 Europeans and 14 462 Asians. A total of 41 genome‐wide significant loci were found, 20 of which were novel discoveries. Of the 41 loci, 90% were replicated in an independent sample of European participants from the UK Biobank. Meta‐analysis of the CREAM and UK Biobank results revealed that the five loci most strongly associated with corneal curvature were: rs4074961 (*RSPO1*), rs12702376 (*HUS1*), rs9506725 (*FGF9*), rs1800813 (*PDGFRA*) and rs2630445 (*HDAC11*‐*FBLN2*). The effect sizes and directions of the corneal curvature GWAS lead variants were consistent across European and East Asian ancestries. However, three loci (*HDAC11*‐*FBLN2*, *ADAMTS19*‐*CASY3* and *FGF9*) were uniquely associated in individuals of European ancestry, while two were uniquely associated in individuals of East Asian ancestry (*RBP3* and *CMPK1‐STIL*). Variants rs2240776 in the vicinity of *EMX2*‐*EMX2OS* and rs7672919 in *NCAPG* were identified in single ancestry GWAS analysis of East Asians. Notably, 8 of the 41 lead variants associated with corneal curvature were also associated with axial length but not refractive error: in other words, these eight variants influenced corneal curvature and axial length in tandem to regulate ‘eye size’. An additional 15 of the 41 lead variants were associated with refractive error, while the remaining 18 variants were not definitively associated with either axial length or refractive error, likely due to limited statistical power. Follow‐up gene‐based analyses highlighted the genes *ANKRD65*, *PEAR1*, *ASB1*, *GMDS*, *EMX2OS* and *HM13*‐*AS1*, and implicated two molecular pathways relating to the extracellular matrix, as well as other pathways involved in eye development, organism development and growth, connective tissue cartilage and glycosylation protein activity.

Building on the GWAS of axial length reported by CREAM more than a decade ago (Cheng et al., 2013), a recent CREAM study by Jiang et al. (2023) tested genetic variants for an association with axial length in a sample of 19 420 adults of European, non‐Hispanic/Latino, Asian and African ancestry from the Genetic Epidemiology Research on Adult Health and Aging (GERA) cohort. Replication of the findings was carried out in the original CREAM GWAS sample of East Asian and European ancestry. Jiang et al. identified 16 loci associated with axial length at genome‐wide significance, 5 of which were novel: rs57718990 (*SLC25A12*), rs1353386 (*BMP3*), rs140405296 (*RGR*), rs58514548 (*RBFOX1*) and rs55754534 (*MYO5B*) (Table 3). Additionally, a SNP at the *PRSS56* locus was associated with axial length specifically in non‐Hispanic white participants. Only two of the five novel variants were available for testing in the CREAM cohort; rs1353386 was convincingly replicated while rs55754534 was nominally significant. All 17 of the axial length‐associated variants identified in the GERA cohort were also associated with refractive error, and there was a high negative correlation (*r* = −0.86) in their effect sizes for the two traits. Indeed, the genome‐wide genetic correlation between axial length and refractive error in the GERA sample was similarly high (*r*_g_ = −0.83), as was that for axial length versus myopia (*r*_g_ = +0.80). These results suggest a widespread shared genetic contribution to these traits. Pathway analysis highlighted roles relating to cranial skeletal development and the formation of elastic fibres, consistent with findings from previous work (Ouyang et al., 2019).

Emmetropic eyes are characterized by coordinated scaling of the various ocular components, with a match between corneal curvature and axial length. A CREAM study by Plotnikov et al. (2021) found that corneal curvature and axial length were linearly related in emmetropic children, despite these children's eyes having a remarkably wide range of axial lengths (21–25 mm). The authors carried out a GWAS for corneal curvature in a sample of 22 180 emmetropic adults, using the corneal curvature phenotype as a proxy for eye size—based on the characteristic scaling of axial length and corneal curvature in emmetropes. A total of 32 genetic variants were identified in the discovery stage. The most strongly associated variants were located in the genes *WNT7* (rs73175081), *FGF9* (rs9506727), *RSPO1* (rs4074961) and *PDGFRA* (rs12503971); these variants were in high linkage disequilibrium (LD) with known refractive error or eye size‐associated GWAS variants at these loci. However, 14 of the variants were situated in genomic regions not previously linked to eye size or refractive error, nearby the genes: *ARHGAP32*, *INTS11*, *LOC100506532*, *LOC101928278*, *LOC105369896*, *LOC105375907*, *LOC105375911*, *LOXL1*, *NAV3*, *PI15*, *PIEZO2*, *PKD1L1*, *PPP2R3A* and *RBL2*. These SNPs were validated specifically for eye size rather than refractive error by deriving a polygenic score for eye size constructed using the 32 genome‐wide significant SNPs. The polygenic score for eye size explained 3.5% of the variance in corneal curvature and 2.0% of the variance in axial length in an independent sample of emmetropic children, while the same polygenic score had no association with corneal curvature or axial length in an equivalent sample of ametropic children. The genetic correlation between eye size and refractive error was close to zero (*r*_g_ = 0.00 ± 0.06), which led Plotnikov et al. to conclude that distinct sets of genes control ‘normal’, coordinated eye growth versus the imbalanced axial elongation that occurs during refractive error development.

## 8 PREDICTING WHICH INDIVIDUALS ARE AT RISK OF HIGH MYOPIA

Polygenic scores quantifying a person's genetic susceptibility to myopia or hyperopia have been proposed to identify children at elevated risk of high myopia. A recent CREAM study by Clark et al. (2023) reported a new polygenic score that had a level of accuracy in the European population close to that required for clinical use. The study used the multi‐trait analysis of GWAS (MTAG) method to meta‐analyse four European samples, which boosted precision when estimating the effect sizes of genetic variants. Accuracy assessments were conducted in an independent sample of 6000 European participants as well as in smaller East Asian, South Asian and African ancestry samples from the UK Biobank. Children from the longitudinal Generation R and ALSPAC cohorts were also used as replication samples. The new polygenic score explained 19% of the variance in refractive error in the European ancestry replication sample, but performance was far worse in the three other ancestries, especially in those of African ancestry. For individuals of European ancestry, the AUROC was 0.77 when predicting high myopia ≤−6.00D and AUROC = 0.78 for high myopia ≤−5.00 D. Persons with a polygenic score within the lowest 5% of the population had a six‐ to sevenfold increased risk of high myopia compared to the remaining 95% of the population. High myopia prediction was equally effective in 13‐year‐old children from the Generation R cohort (AUROC = 0.78). Notably, however, the accuracy was below AUROC = 0.70 in the other three ancestries. As well as its potential for predicting the risk of myopia in children, Clark et al. also tested whether the new polygenic score could be used in older adults to predict patients at elevated risk of myopic macular degeneration (MMD). Despite a strong association between the polygenic score and severe grades of MMD when the relationship was examined in isolation, the polygenic score was not predictive of severe MMD risk once the patient's refractive error was accounted for.

## 9 CONCLUSION

Research carried out by the CREAM consortium over the past 5 years has continued to enhance knowledge regarding the aetiology of myopia. These advances have been achieved by research strategies that include: studying cohorts of more diverse ancestry; identifying novel genetic loci—especially rare variants and variants located in non‐coding regions of the genome; exploring shared genetic effects across myopia endophenotypes; dissection of specific genetic pathways implicated in myopia development and emmetropization; and improving methods for predicting high myopia risk. In particular, recent efforts from the CREAM Consortium have revealed several candidate genes that deserve attention as potential therapeutic targets, such as *SIX6*, *CRX*, *PER3*, *PDCD6IP*, *MAPT*, *CHST6*, *GRHL2*, *USH2A*, *P4HTM*, *COL4A4*, *ATM* and *DDIT4*. In addition, a polygenic risk score with accuracy for detecting children at risk of high myopia (AUC = 0.78) was reported, which approaches the level of accuracy required for use in clinical screening. Nevertheless, further work is needed to explain the remaining ‘missing heritability’ of refractive error and to better clarify the genetic architecture of myopia, such as the total contribution of rare variants. There is still a major gap between genetic discoveries in myopia research and their translation into improved patient care. Discovering the functional impact of genetic variants, understanding how genes and lifestyle factors interact to cause either early‐onset or late‐onset high myopia, and evaluating whether genetic differences explain inter‐individual myopia control treatment efficacy are exciting areas for future research.

## FUNDING INFORMATION

Supported by UK Research and Innovation (UKRI) under the UK government's Horizon Europe funding guarantee (grant number EP/Y032292/1). Funded by the European Union (Project 101119501—MyoTreat—HORIZON‐MSCA‐2022‐DN‐01). Views and opinions expressed are, however, those of the author(s) only and do not necessarily reflect those of the European Union or UKRI. Neither the European Union nor the granting authority can be held responsible for them.

## Data Availability

No new data were generated as part of this work.

## References

[aos70025-bib-0001] Aldahmesh, M.A. , Khan, A.O. , Hijazi, H. & Alkuraya, F.S. (2013) Homozygous truncation of SIX6 causes complex microphthalmia in humans. Clinical Genetics, 84, 198–199. 23167593 10.1111/cge.12046

[aos70025-bib-0002] Brown‐Panton, C.A. , Sabour, S. , Zoidl, G.S.O. , Zoidl, C. , Tabatabaei, N. & Zoidl, G.R. (2023) Gap junction delta‐2b (gjd2b/Cx35.1) depletion causes hyperopia and visual‐motor deficiencies in the zebrafish. Frontiers in Cell and Developmental Biology, 11, 1150273. 36936688 10.3389/fcell.2023.1150273 PMC10017553

[aos70025-bib-0003] Chen, C. , An, G. , Yu, X. , Wang, S. , Lin, P. , Yuan, J. et al. (2024) Screening mutations of the monogenic syndromic high myopia by whole exome sequencing from MAGIC project. Investigative Ophthalmology & Visual Science, 65, 9. 10.1167/iovs.65.2.9 PMC10851780 38315492

[aos70025-bib-0004] Cheng, C.‐Y. , Schache, M. , Ikram, M.K. , Young, T.L. , Guggenheim, J.A. , Vitart, V. et al. (2013) Nine loci for ocular axial length identified through genome‐wide association studies, including shared loci with refractive error. American Journal of Human Genetics, 93, 264–277. 24144296 10.1016/j.ajhg.2013.06.016 PMC3772747

[aos70025-bib-0005] Clark, R. , Lee, S.S. , Du, R. , Wang, Y. , Kneepkens, S.C.M. , Charng, J. et al. (2023) A new polygenic score for refractive error improves detection of children at risk of high myopia but not the prediction of those at risk of myopic macular degeneration. eBioMedicine, 91, 104551. 37055258 10.1016/j.ebiom.2023.104551 PMC10203044

[aos70025-bib-0006] Cuellar‐Partida, G. , Williams, K.M. , Yazar, S. , Guggenheim, J.A. , Hewitt, A.W. , Williams, C. et al. (2017) Genetically low vitamin D concentrations and myopic refractive error: a Mendelian randomization study. International Journal of Epidemiology, 46, 1882–1890. 28586461 10.1093/ije/dyx068 PMC5837568

[aos70025-bib-0007] Fan, Q. , Guo, X. , Tideman, J.W. , Williams, K.M. , Yazar, S. , Hosseini, S.M. et al. (2016) Childhood gene‐environment interactions and age‐dependent effects of genetic variants associated with refractive error and myopia: the CREAM consortium. Scientific Reports, 6, 25853. 27174397 10.1038/srep25853 PMC4865831

[aos70025-bib-0008] Fan, Q. , Pozarickij, A. , Tan, N.Y.Q. , Guo, X. , Verhoeven, V.J.M. , Vitart, V. et al. (2020) Genome‐wide association meta‐analysis of corneal curvature identifies novel loci and shared genetic influences across axial length and refractive error. Communications Biology, 3, 133. 32193507 10.1038/s42003-020-0802-y PMC7081241

[aos70025-bib-0009] Fan, Q. , Verhoeven, V.J.M. , Wojciechowski, R. , Barathi, V.A. , Hysi, P.G. , Guggenheim, J.A. et al. (2016) Meta‐analysis of gene‐environment‐wide association scans accounting for education level identifies additional loci for refractive error. Nature Communications, 7, 11008. 10.1038/ncomms11008 PMC4820539 27020472

[aos70025-bib-0010] Freund, C.L. , Wang, Q.L. , Chen, S. , Muskat, B.L. , Wiles, C.D. , Sheffield, V.C. et al. (1998) De novo mutations in the CRX homeobox gene associated with Leber congenital amaurosis. Nature Genetics, 18, 311–312. 9537410 10.1038/ng0498-311

[aos70025-bib-0011] Furukawa, T. , Morrow, E.M. & Cepko, C.L. (1997) Crx, a novel otx‐like homeobox gene, shows photoreceptor‐specific expression and regulates photoreceptor differentiation. Cell, 91, 531–541. 9390562 10.1016/s0092-8674(00)80439-0

[aos70025-bib-0012] Guggenheim, J.A. , Clark, R. , Cui, J. , Terry, L. , Patasova, K. , Haarman, A.E.G. et al. (2022) Whole exome sequence analysis in 51,624 participants identifies novel genes and variants associated with refractive error and myopia. Human Molecular Genetics, 31, 1909–1919. 35022715 10.1093/hmg/ddac004 PMC9169456

[aos70025-bib-0013] Haarman, A.E.G. , Enthoven, C.A. , Tedja, M.S. , Polling, J.R. , Tideman, J.W.L. , Keunen, J.E.E. et al. (2021) Phenotypic consequences of the GJD2 risk genotype in myopia development. Investigative Ophthalmology & Visual Science, 62, 16. 10.1167/iovs.62.10.16 PMC8375003 34406332

[aos70025-bib-0014] Haarman, A.E.G. , Enthoven, C.A. , Tideman, J.W.L. , Tedja, M.S. , Verhoeven, V.J.M. & Klaver, C.C.W. (2020) The complications of myopia: a review and meta‐analysis. Investigative Ophthalmology & Visual Science, 61, 49. 10.1167/iovs.61.4.49 PMC7401976 32347918

[aos70025-bib-0015] Haarman, A.E.G. , Tedja, M.S. , Meester‐Smoor, M.A. , Kaprio, J. , Mackey, D.A. , Guggenheim, J.A. et al. (2021) Consortium for Refractive Error and Myopia (CREAM): vision, mission, and accomplishments. In: Prakash, G. & Iwata, T. (Eds.) Advances in vision research, volume III: Genetic eye research around the globe. Singapore: Springer, pp. 381–407.

[aos70025-bib-0016] Holden, B.A. , Fricke, T.R. , Wilson, D.A. , Jong, M. , Naidoo, K.S. , Sankaridurg, P. et al. (2016) Global prevalence of myopia and high myopia and temporal trends from 2000 through 2050. Ophthalmology, 123, 1036–1042. 26875007 10.1016/j.ophtha.2016.01.006

[aos70025-bib-0017] Hysi, P.G. , Choquet, H. , Khawaja, A.P. , Wojciechowski, R. , Tedja, M.S. , Yin, J. et al. (2020) Meta‐analysis of 542,934 subjects of European ancestry identifies new genes and mechanisms predisposing to refractive error and myopia. Nature Genetics, 52, 401–407. 32231278 10.1038/s41588-020-0599-0 PMC7145443

[aos70025-bib-0018] Jiang, C. , Melles, R.B. , Yin, J. , Fan, Q. , Guo, X. , Cheng, C.Y. et al. (2023) A multiethnic genome‐wide analysis of 19,420 individuals identifies novel loci associated with axial length and shared genetic influences with refractive error and myopia. Frontiers in Genetics, 14, 1113058. 37351342 10.3389/fgene.2023.1113058 PMC10282939

[aos70025-bib-0019] Kiefer, A.K. , Tung, J.Y. , Do, C.B. , Hinds, D.A. , Mountain, J.L. , Francke, U. et al. (2013) Genome‐wide analysis points to roles for extracellular matrix remodeling, the visual cycle, and neuronal development in myopia. PLoS Genetics, 9, e1003299. 23468642 10.1371/journal.pgen.1003299 PMC3585144

[aos70025-bib-0020] Li, Q. , Wojciechowski, R. , Simpson, C.L. , Hysi, P.G. , Verhoeven, V.J.M. , Ikram, M.K. et al. (2015) Genome‐wide association study for refractive astigmatism reveals genetic co‐determination with spherical equivalent refractive error: the CREAM consortium. Human Genetics, 134, 131–146. 25367360 10.1007/s00439-014-1500-y PMC4291519

[aos70025-bib-0021] Li, Y. , Lu, Y. , Du, K. , Yin, Y. , Hu, T. , Fu, Q. et al. (2022) RNA‐sequencing analysis reveals the long noncoding RNA profile in the mouse myopic retina. Frontiers in Genetics, 13, 1014031. 36313450 10.3389/fgene.2022.1014031 PMC9606684

[aos70025-bib-0022] Morgan, I.G. , Wu, P.‐C. , Ostrin, L.A. , Tideman, J.W.L. , Yam, J.C. , Lan, W. et al. (2021) IMI risk factors for myopia. Investigative Ophthalmology & Visual Science, 62, 3. 10.1167/iovs.62.5.3 PMC8083079 33909035

[aos70025-bib-0023] Musolf, A.M. , Haarman, A.E.G. , Luben, R.N. , Ong, J.S. , Patasova, K. , Trapero, R.H. et al. (2023) Rare variant analyses across multiethnic cohorts identify novel genes for refractive error. Communications Biology, 6, 6. 36596879 10.1038/s42003-022-04323-7 PMC9810640

[aos70025-bib-0024] Ohno‐Matsui, K. , Wu, P.‐C. , Yamashiro, K. , Vutipongsatorn, K. , Fang, Y. , Cheung, C.M.G. et al. (2021) IMI pathologic myopia. Investigative Ophthalmology & Visual Science, 62, 5. 10.1167/iovs.62.5.5 PMC8083114 33909033

[aos70025-bib-0025] Ouyang, X. , Han, Y. , Xie, Y. , Wu, Y. , Guo, S. , Cheng, M. et al. (2019) The collagen metabolism affects the scleral mechanical properties in the different processes of scleral remodeling. Biomedicine & Pharmacotherapy, 118, 109294. 31404770 10.1016/j.biopha.2019.109294

[aos70025-bib-0026] Patasova, K. , Haarman, A.E.G. , Musolf, A.M. , Mahroo, O.A. , Rahi, J.S. , Falchi, M. et al. (2022) Association analyses of rare variants identify two genes associated with refractive error. PLoS One, 17, e0272379. 36137074 10.1371/journal.pone.0272379 PMC9499304

[aos70025-bib-0027] Plotnikov, D. , Cui, J. , Clark, R. , Wedenoja, J. , Pärssinen, O. , Tideman, J.W.L. et al. (2021) Genetic variants associated with human eye size are distinct from those conferring susceptibility to myopia. Investigative Ophthalmology & Visual Science, 62, 24. 10.1167/iovs.62.13.24 PMC8556552 34698770

[aos70025-bib-0028] Quint, W.H. , Tadema, K.C.D. , de Vrieze, E. , Lukowicz, R.M. , Broekman, S. , Winkelman, B.H.J. et al. (2021) Loss of gap junction delta‐2 (GJD2) gene orthologs leads to refractive error in zebrafish. Communications Biology, 4, 676. 34083742 10.1038/s42003-021-02185-z PMC8175550

[aos70025-bib-0029] Sanfilippo, P.G. , Hewitt, A.W. , Hammond, C.J. & Mackey, D.A. (2010) The heritability of ocular traits. Survey of Ophthalmology, 55, 561–583. 20851442 10.1016/j.survophthal.2010.07.003

[aos70025-bib-0030] Shah, R.L. , Li, Q. , Zhao, W. , Tedja, M.S. , Tideman, J.W.L. , Khawaja, A.P. et al. (2018) A genome‐wide association study of corneal astigmatism: the CREAM consortium. Molecular Vision, 24, 127–142. 29422769 PMC5800430

[aos70025-bib-0031] Swain, P.K. , Chen, S. , Wang, Q.L. , Affatigato, L.M. , Coats, C.L. , Brady, K.D. et al. (1997) Mutations in the cone‐rod homeobox gene are associated with the cone‐rod dystrophy photoreceptor degeneration. Neuron, 19, 1329–1336. 9427255 10.1016/s0896-6273(00)80423-7

[aos70025-bib-0032] Swierkowska, J. , Vishweswaraiah, S. , Mrugacz, M. , Radhakrishna, U. & Gajecka, M. (2022) Differential methylation of microRNA encoding genes may contribute to high myopia. Frontiers in Genetics, 13, 1089784. 36685896 10.3389/fgene.2022.1089784 PMC9847511

[aos70025-bib-0033] Tedja, M.S. , Haarman, A.E.G. , Meester‐Smoor, M.A. , Kaprio, J. , Mackey, D.A. , Guggenheim, J.A. et al. (2019) IMI – myopia genetics report. Investigative Ophthalmology & Visual Science, 60, M89–M105. 30817828 10.1167/iovs.18-25965 PMC6892384

[aos70025-bib-0034] Tedja, M.S. , Swierkowska‐Janc, J. , Enthoven, C.A. , Meester‐Smoor, M.A. , Hysi, P.G. , Felix, J.F. et al. (2025) A genome‐wide scan of non‐coding RNAs and enhancers for refractive error and myopia. Human Genetics, 144, 67–91. 39774722 10.1007/s00439-024-02721-x PMC11754329

[aos70025-bib-0035] Tedja, M.S. , Wojciechowski, R. , Hysi, P.G. , Eriksson, N. , Furlotte, N.A. , Verhoeven, V.J.M. et al. (2018) Genome‐wide association meta‐analysis highlights light‐induced signaling as a driver for refractive error. Nature Genetics, 50, 834–848. 29808027 10.1038/s41588-018-0127-7 PMC5980758

[aos70025-bib-0036] Tideman, J.W.L. , Pärssinen, O. , Haarman, A.E.G. , Khawaja, A.P. , Wedenoja, J. , Williams, K.M. et al. (2021) Evaluation of shared genetic susceptibility to high and low myopia and hyperopia. JAMA Ophthalmology, 139, 601–609. 33830181 10.1001/jamaophthalmol.2021.0497 PMC8033508

[aos70025-bib-0037] van der Sande, E. , Haarman, A.E.G. , Quint, W.H. , Tadema, K.C.D. , Meester‐Smoor, M.A. , Kamermans, M. et al. (2022) The role of GJD2(Cx36) in refractive error development. Investigative Ophthalmology & Visual Science, 63, 5. 10.1167/iovs.63.3.5 PMC8934558 35262731

[aos70025-bib-0038] Verhoeven, V.J. , Hysi, P.G. , Saw, S.M. , Vitart, V. , Mirshahi, A. , Guggenheim, J.A. et al. (2012) Large scale international replication and meta‐analysis study confirms association of the 15q14 locus with myopia. The CREAM Consortium. Human Genetics, 131, 1467–1480. 22665138 10.1007/s00439-012-1176-0 PMC3418496

[aos70025-bib-0039] Verhoeven, V.J.M. , Hysi, P.G. , Wojciechowski, R. , Fan, Q. , Guggenheim, J.A. , Hohn, R. et al. (2013) Genome‐wide meta‐analyses of multiancestry cohorts identify multiple new susceptibility loci for refractive error and myopia. Nature Genetics, 45, 314–318. 23396134 10.1038/ng.2554 PMC3740568

[aos70025-bib-0040] Wang, Y.M. , Sy, L. , Zhang, X.J. , Chen, L.J. , Pang, C.P. & Yam, J.C. (2022) Myopia genetics and heredity. Children, 9, 382. 35327754 10.3390/children9030382 PMC8947159

[aos70025-bib-0041] Wang, Y.Y. , Chen, L.J. , Tham, C.C. , Yam, J.C. & Pang, C.P. (2025) Genes for childhood myopia. Asia‐Pacific Journal of Ophthalmology, 14, 100139. 39814143 10.1016/j.apjo.2025.100139

[aos70025-bib-0042] Yuan, J. , Qiu, R. , Wang, Y. , Chen, Z.J. , Sun, H. , Dai, W. et al. (2024) Exome‐wide genetic risk score (ExGRS) to predict high myopia across multi‐ancestry populations. Communications Medicine, 4, 280. 39738800 10.1038/s43856-024-00718-1 PMC11685959

